# The impact of India-ASEAN free trade agreement on trade flows: An application of augmented gravity model

**DOI:** 10.1371/journal.pone.0350036

**Published:** 2026-06-03

**Authors:** Prajakta Arote, Hastimal Sagara, Jadhav Chakradhar, Guruprasad Desai, Jadhav Pravin, Din Bandhu

**Affiliations:** 1 GLS University, Ahmedabad, Gujarat, India; 2 Centre for Economic and Social Studies (CESS), Hyderabad, Telangana, India; 3 Department of Commerce, Manipal Academy of Higher Education, Manipal, India; 4 Institute of Infrastructure, Technology, Research and Management (IITRAM), Ahmedabad, Gujarat, India; 5 Galgotias University, Greater Noida, Uttar Pradesh, India; Islamia University of Bahawalpur, PAKISTAN

## Abstract

This study investigates the impact of the India-ASEAN Free Trade Agreement (FTA) on bilateral trade flows through an augmented gravity model. Employing panel data from 1990 to 2022 with 330 observations, the analysis incorporates key determinants such as GDP and population of India and ASEAN countries, tariff rates, geographic distance, historical ties, and levels of democracy, trade openness, and globalization. To address heteroskedasticity and cross-sectional dependency, we utilize Generalized Least Squares (GLS) estimations. Our analysis examines both export and import volumes between India and ASEAN nations. Results from Ordinary Least Squares (OLS) and GLS estimations indicate that ASEAN countries’ GDP positively influences trade volumes, while India’s GDP demonstrates mixed effects. Larger populations in both India and ASEAN countries significantly contribute to trade. Tariff rates and geographic distance are found to have a negative effect on both exports and imports. Interestingly, shared colonial history and language positively impact trade, whereas a common border yields mixed effects across the models. Control variables reveal that trade openness, democracy levels, and globalization have a positive and significant effect on India-ASEAN trade flows. The FTA dummy is positive and highly significant, confirming that AIFTA has substantially increased India’s bilateral trade with ASEAN. Based on these findings, we suggest that enhancing economic growth, mitigating geographic barriers, leveraging historical ties, and promoting trade openness and regional integration are essential strategies to bolster trade between India and ASEAN countries.

## 1. Introduction

The economic relationship between India and the Association of Southeast Asian Nations (ASEAN) has gained increasing significance, driven by the rapid development of both regions. Since the 1990s, India has sought to deepen its economic integration with ASEAN, initially through the ‘Look East Policy’ and subsequently through the upgraded ‘Act East Policy’ in 2014. A major milestone in this evolving relationship was the ASEAN-India Free Trade Agreement (AIFTA), signed in 2009 and implemented in 2010. The agreement aimed to stimulate bilateral trade by progressively reducing tariffs on a significant share of goods traded between the two regions. As a result, ASEAN has become India’s fourth-largest trading partner, constituting nearly 11 percent of India’s total global trade (Source: https://pib.gov.in/PressReleaseIframePage.aspx?PRID=2020351).

However, the trade outcomes under AIFTA have not been wholly favourable for India. While overall trade volumes have expanded, the trade balance has shifted markedly in favor of ASEAN, resulting in a substantial increase in India’s trade deficit with the region. This growing deficit, along with India’s limited export expansion to ASEAN, has raised questions about the efficacy of AIFTA and other free trade agreements (FTAs) India has pursued. These concerns eventually led to India’s decision to abstain from the Regional Comprehensive Economic Partnership (RCEP) in 2019, largely due to apprehensions that increased imports would outweigh the potential gains from export growth. In this context, reassessing the trade and economic relationship between India and ASEAN, particularly the effectiveness of AIFTA in supporting Indian exports, has become imperative. This study addresses this need by examining the impact of AIFTA on India’s goods exports to ASEAN, using an augmented gravity model applied to panel data from 1990–2022. The gravity model, widely recognized in economic literature, offers a robust framework for analysing the economic and welfare impacts of FTAs.

This study contributes uniquely to the literature on India-ASEAN trade by incorporating various economic and noneconomic factors, acknowledging their critical role that shape regional integration outcomes. Additionally, it improves upon previous analyses by including the country and time fixed effect which accounts for Multilateral Trade Resistance (MTR) term in the gravity model to address potential estimation biases that may have affected earlier studies. Further enhancing the model, we incorporate indicators of globalization and democracy levels for India and its ASEAN trading partners, providing a comprehensive perspective on how these political and economic factors influence trade flows, which is ignored in the previous literature.

This paper thus makes several key contributions: first, it strengthens the theoretical rigor and empirical accuracy of India-ASEAN trade analysis by including tariff measures, and the incorporating the MTR term. Second, it expands the understanding of non-traditional determinants by considering globalization and democracy levels as influential variables. Finally, the study offers evidence-based recommendations on optimizing trade and policy frameworks to bolster India’s trade relationship with ASEAN under the AIFTA.

The remainder of the paper is organized as follows: Section 2 presents an overview of India-ASEAN trade flows from 1990 to 2022. Section 3 reviews the relevant literature on India-ASEAN trade agreements, followed by a background discussion on AIFTA in Section 4. Section 5 introduces the methodology, variables, and data sources, while Section 6 presents the empirical analysis and interpretation of results. The final section concludes with key findings and discusses their implications for future trade policy.

## 2. Overview of Indian- ASEAN trade flows

Table 1 presents India’s exports to ASEAN member countries from 1990 to 2022, highlighting a significant upward trend. Total exports increased from $871.538 million in 1990 to $44,047.192 million in 2022. Following the implementation of the ASEAN-India Free Trade Agreement (AIFTA) in 2009, India’s exports to most ASEAN countries—except Laos—have risen considerably. Key ASEAN members, such as Singapore, Malaysia, and Indonesia, have consistently served as major destinations for Indian exports. For instance, India’s exports to Singapore surged from $322.273 million in 1990 to $11,830.795 million in 2022, establishing Singapore as a crucial trade hub within the region. Substantial growth is also observed in exports to other markets such as Vietnam and the Philippines. The adoption of India’s Look East Policy in the early 2000s has played an instrumental role in shaping foreign trade strategies, thus strengthening trade relationships with ASEAN countries.

**Table 1:** India’s Exports to ASEAN Member Countries- 1990-2022 *(values in million US$).*

Year	Brunei	Cambodia	Indonesia	Lao	Malaysia	Myanmar	Philippines	Singapore	Thailand	Viet Na	ASEAN
1990	0.669	0.136	108.132	0.030	149.212	1.851	28.028	322.273	244.250	16.957	871.538
1995	7.221	2.131	660.383	0.310	391.729	30.084	143.756	889.522	471.230	123.985	2720.352
2000	3.057	7.886	390.371	3.506	530.951	43.185	174.192	787.048	525.237	195.393	2660.827
2005	4.395	21.350	1390.066	6.540	1143.775	117.246	482.110	5427.555	1059.267	633.465	10285.769
2010	21.239	61.046	4557.083	8.180	3555.312	272.579	801.607	9066.230	2139.581	2475.596	22958.453
2015	30.392	145.346	2868.880	51.264	4892.061	859.973	1304.346	7805.084	3113.562	5357.213	26428.121
2020	60.051	144.043	4363.742	27.874	6194.006	837.624	1416.026	8295.020	3777.064	4500.549	29615.999
2022	68.810	227.355	9866.614	16.900	7190.535	765.164	2160.846	11830.795	6039.395	5880.778	44047.192

Source: WITS.

Table 2 presents India’s imports from ASEAN member countries, showing a substantial increase from $1,318.036 million in 1990 to $89,305.637 million in 2022. Indonesia, Malaysia, Thailand, and Singapore have emerged as major import sources for India, with import values rising significantly over time. Imports from Indonesia, for example, grew from $80.209 million in 1990 to $28,665.341 million in 2022, making it one of India’s largest trading partners within ASEAN. Similarly, imports from Malaysia increased from $528.868 million in 1990 to $13,542.358 million in 2022, primarily driven by strong trade in sectors such as palm oil, electronics, and machinery. Singapore also became a significant partner, with imports rising from $490.982 million in 1990 to $24,418.725 million in 2022. Other ASEAN countries, including Vietnam and the Philippines, have also shown notable growth in trade with India, albeit from a lower base. This trend indicates the increasing interdependence between India and ASEAN, which is expected to strengthen further through initiatives such as the ASEAN-India Free Trade Agreement (AIFTA) and the Act East Policy.

**Table 2:** India’s Imports from ASEAN Member Countries- 1990-2022 *(values in million US$).*

Year	Brunei	Cambodia	Indonesia	Lao	Malaysia	Myanmar	Philippines	Singapore	Thailand	Vietnam	ASEAN
1990	0.004	0.663	80.209	0.263	528.868	91.604	4.497	490.982	63.758	57.189	1318.036
1995	0.036	0.341	460.089	NA	896.819	161.989	21.412	853.340	169.299	15.456	2578.780
2000	0.184	1.270	985.839	NA	1311.030	178.499	68.256	1401.560	339.212	13.541	4299.391
2005	0.832	0.425	3018.949	0.072	2435.996	489.162	203.196	3159.416	1196.597	127.378	10632.024
2010	207.130	7.638	9695.329	20.117	5995.904	1122.147	394.450	7263.136	3940.819	993.512	29640.184
2015	607.783	42.989	13902.025	142.955	9559.921	1016.301	518.188	7395.998	5650.145	2680.089	41516.392
2020	422.894	38.186	12020.795	2.075	7378.041	575.594	505.658	12306.747	5223.762	5564.634	44038.387
2022	317.254	120.435	28665.341	57.533	13542.358	1032.316	896.446	24418.725	11250.487	9004.743	89305.637

Source: WITS. Note: NA = Data is not available.

## 3. Review of literature

### 3.1. Proliferation of RTAs and the WTO debate

The proliferation of Regional Trade Agreements (RTAs) since the 1990s has sparked significant debate in international trade policy, particularly regarding their alignment with the World Trade Organization (WTO) system [1]. Initially, discussions focused on whether RTAs acted as “building blocks” or “stumbling blocks” for the WTO. Recently, however, the emphasis has shifted towards ensuring that RTAs complement the multilateral framework of the WTO, rather than undermining it. Three main areas of contention persist: (1) many RTAs significantly exclude agriculture from liberalization, despite its critical role in global trade; (2) intricate and restrictive rules of origin within RTAs often complicate trade processes and can distort trade flows; and (3) the varied treatment of trade remedy laws across RTAs leads to inconsistencies with WTO rules.

The ongoing debate regarding RTA compatibility with WTO principles has prompted various proposals for reforming GATT Article XXIV. The literature highlights a need to balance reforms with the specific needs of developing countries, while addressing potential impacts on ACP (African, Caribbean, and Pacific) countries [2,3]. Proposals such as the Kemp-Wan compatibility criterion are suggested as alternatives to the current “catch me if you can” approach, potentially fostering a more cohesive multilateral trade environment. This approach acknowledges the role of both multilateral and regional efforts—such as those by the US—in aligning RTAs with the WTO framework, viewing these efforts as complementary to, rather than replacements for, the WTO’s mandate [1]. This body of literature underscores the need for a multi-faceted approach involving both multilateral reforms and unilateral/regional initiatives to harmonize RTAs with WTO principles. Future research could explore the effectiveness of these proposed mechanisms to further strengthen the global trading system.

### 3.2. Impact of RTAs on trade flows, economic growth, and trade dynamics

[4] investigated the effects of RTAs on trade creation and trade diversion in the Asia-Pacific region using a gravity model. Their study examined whether RTAs within this region fostered trade among member countries at the expense of non-members. Specifically, they compared RTAs such as ASEAN, CER, APEC, MERCOSUR, and NAFTA, identifying ASEAN and CER as “trade-creating” agreements that promote trade inclusively with both members and non-members, enhancing welfare. In contrast, APEC, MERCOSUR, and NAFTA were characterized as “trade-diverting,” favoring intra-bloc trade over trade with external partners, potentially diminishing welfare. The study attributes these disparities to differences in the liberalization levels and trade concession structures among RTAs. ASEAN and CER’s broader reduction of trade barriers, including for non-members, exemplifies a more outward-oriented trade policy, while APEC, MERCOSUR, and NAFTA reflect a more inward-looking stance. By applying a gravity model and including comprehensive cost factors, the authors provide valuable insights into the trade and welfare effects of these RTAs, highlighting the significance of trade creation and trade diversion in regional trade agreements.

[5] examined trade complementarities between India and ASEAN countries using the Trade Intensity Index (TII) and Revealed Comparative Advantage (RCA) Index. The study identified substantial trade complementarities, suggesting strong potential for enhanced economic collaboration. India’s trade intensity with ASEAN countries exceeded unity, signalling robust trade ties, while RCA analysis highlighted India’s comparative advantage in sectors like agriculture, chemicals, and textiles. Meanwhile, ASEAN nations demonstrated strengths in machinery, transport equipment, and electronics. The study also revealed competitive overlaps in textiles, posing challenges under regional agreements. Chandran concluded that while tariff reductions under the India-ASEAN Free Trade Agreement (AIFTA) may initially impact India’s exports, medium-term productivity gains could strengthen economic ties further, emphasizing the need for targeted synergies to maximize mutual benefits.

### 3.3. India-ASEAN free trade agreement

Research on the India-ASEAN Free Trade Agreement (AIFTA) predominantly examines its impact on trade dynamics between India and ASEAN countries [6–19]. Studies, such as [8], have indicated that AIFTA led to a deterioration in India’s trade balance with ASEAN, with a declining trade surplus turning negative post-2012, influenced by the global economic slowdown. [20] used a gravity model to assess the AIFTA’s impact on agricultural trade, suggesting that the agreement has a pure trade-creating effect, benefiting the agricultural sector from further liberalization.

[21] evaluated trade creation and diversion effects under IAFTA, noting that while the agreement fosters bilateral trade, it generates more import than export creation, a potential target for policy intervention. [22] examined trade specialization, finding that India is gradually transitioning towards a competitive and specialized economy but still faces significant competition from ASEAN members. This study underscores the necessity of technological advancement and diversification to strengthen trade resilience under AIFTA.

[23,24] discussed AIFTA-related issues, noting inconclusive evidence on its overall benefits to India. They argue that while the agreement has potential, particularly in services, future assessments are necessary to determine its long-term value. Overall, the literature suggests that AIFTA has spurred trade creation but has also introduced challenges, including trade deficits and heightened competition. Policymakers are encouraged to address these issues to enhance the agreement’s benefits [25–27].

The current study builds on this body of work by offering a comprehensive analysis of the India-ASEAN Free Trade Agreement, using an extended time series of data and advanced econometric techniques. While prior studies have often overlooked issues of heteroskedasticity and cross-sectional dependency, this research applies Generalized Least Squares (GLS) methods to deliver more robust and unbiased results. In doing so, this study aims to provide a nuanced understanding of AIFTA’s effects on both exports and imports, contributing valuable insights to the literature on RTAs and trade policy development.

## 4. Data source and variable measurements

The primary objective of this study is to evaluate the impact of the India-ASEAN Free Trade Agreement (AIFTA) on India’s exports and imports over the period from 1990 to 2022. This specific country selection and study period were chosen to meet two main objectives. First, this study aims to analyze the decadal effects of the AIFTA on India’s trade flows with ASEAN countries. Second, the selected variables align with data availability requirements for consistency in empirical analysis. Trade data, including export and import values (in thousands of U.S. dollars), were sourced from the World Integrated Trade Solution (WITS) database, while GDP data for India and ASEAN partner countries, measured in current U.S. dollars, were obtained from the World Bank’s World Development Indicators (WDI) database. Population data, representing the total population of each country, were also gathered from WDI. Trade data are reported in thousands of U.S. dollars in WITS and converted and presented as million US$ in the tables for readability.

Additionally, to capture the liberalization effects, we utilized the Trade Openness Index (TOI) from the WDI, calculated as the ratio of exports and imports to GDP for each respective country. To enhance the analysis, this study incorporates control variables informed by prior research, including the Liberal Democracy Index (LDI) for both exporter and importer countries. LDI data, sourced from the Varieties of Democracy (V-Dem) Institute, represent a scale from 0 to 1, with higher values indicating greater levels of democratic governance. Further, globalization data were retrieved from the WDI database, where a higher score on a 1–100 scale reflects greater globalization. Variables for shared colony, border, and language were collected from the CEPII database to estimate the impact of additional factors on trade flows, as per the augmented gravity model. These variables are treated as binary indicators, set to one if two countries share a particular characteristic and zero otherwise. Our main distance variable, reflecting the distance in kilometres between capital cities of trade partners, was also obtained from CEPII. Prior to analysis, all variables were transformed into natural logarithms to mitigate potential measurement errors. This dataset, balanced across all variables, comprises 330 observations in total. Table 3 provides a detailed overview of variable definitions and data sources.

**Table 3:** Data Sources and Variables Used in the Estimation.

Variable	Source	Expected Sign
Export of India to ASEAN	World Integrated Trade Solutions (WITS)	
Import of India from ASEAN	World Integrated Trade Solutions (WITS)	
Log (GDP_India)	World Bank Development Indicators (WDI)	Positive
Log (GDP_Partner_ASEAN)	World Bank Development Indicators (WDI)	Positive
Log (Popoulation_India)	World Bank Development Indicators (WDI)	Positive
Log (Population_Partner_ASEAN)	World Bank Development Indicators (WDI)	Positive
Log (Tariff)	World Bank Development Indicators (WDI)	Negative
Log (Distance i and j)	CEPII	Negative
Common colony	CEPII	Positive
Common border	CEPII	Positive
Common language	CEPII	Positive
Log (Trade Openness)	World Bank Development Indicators (WDI)	Positive
Log (Level of Democracy_India)	Varieties of Democracy (V-Dem)	Positive
Log (Level of Democracy_Partner)	Varieties of Democracy (V-Dem)	Positive
Log (Globalization)	Varieties of Democracy (V-Dem)	Positive
FTA Dummy (0 = No FTA, 1 = FTA)	Authors’ construction based on AIFTA implementation (2010 onward)	Positive

Source: authors compilation

The study period covers 33 years, from 1990 to 2022. This period was selected to capture the long-term evolution of India–ASEAN trade flows leading up to the ASEAN-India Free Trade Agreement (AIFTA) and the post-FTA adjustment and expansion phase. The early 1990s mark a critical turning point in India’s economic history, as the country undertook wide-ranging economic liberalization measures in 1991. These reforms opened India to global trade and investment, reshaped its tariff structure, and strengthened its linkages with Asian economies. Examining the India–ASEAN trade relationship from this point onward provides important insights into how India’s evolving trade policy orientation has influenced its engagement with Southeast Asia.

## 5. Empirical approach

The present paper employs the panel data augmented gravity model of trade to achieve the study objective. The gravity model of trade has become a dominant framework for understanding bilateral trade flows between countries. This model suggests that the volume of trade between two countries is positively correlated with the size of their economies and negatively correlated with the geographical distance between them [28]. The model’s intuitive appeal and empirical robustness have made it a cornerstone of international trade research. The basic gravity equation can be written as follows:


$$\begin{array}{c} {Trade}_{ij}=\alpha \frac{\overset{\beta_{\text{1}}}{\underset{i}{y}}\overset{\beta_{\text{2}}}{\underset{j}{y}}}{\overset{\beta_{\text{3}}}{\underset{ij}{D}}} \end{array}$$ (1)

Here y_i_ and y_j_ represent the GDPs of the export and import countries, respectively, while D_ij_ denotes the distance between India and its ASEAN trading partners. $\beta_{\text{1}},\;\beta_{\text{2},\;}and\;\beta_{\text{3}}$ are the coefficients of independent variables. By applying the logarithm to the gravity model in Equation (1), we obtain its linear form, as shown in Equation (2).


$$\begin{array}{c} {lntrade}_{it}=\alpha +\beta_{\text{1}}{(GDP}_{it})+{\beta_{\text{2}}(GDP}_{jt})+\beta_{\text{3}}\left({Dist}_{ij}\right)+\mu_{ijt} \end{array}$$ (2)

Initially, the gravity model lacked a rigorous theoretical underpinning and was criticized for its empirical approach without substantial economic justification. Linnemann (1966) [29]  addressed this by linking the model to the Walrasian general equilibrium framework, providing an economic basis for the model. By conceptualizing trade flows as resulting from economic mass (or GDP) and considering barriers like distance, Linnemann established the model as a structural tool within trade theory. [30] introduced a more refined theoretical foundation through the “Armington assumption,” which states that products are differentiated by their country of origin. This assumption allowed the gravity model to explain why trade could exist between similar countries, as goods are not perfect substitutes when produced in different locations. Anderson’s work was significant because it showed how trade patterns could be understood in a general equilibrium context, laying the groundwork for further theoretical advancements.

A major theoretical leap came with [31–33], who introduced a microeconomic foundation for the gravity model by considering price effects within a model of monopolistic competition. Bergstrand integrated price terms into the gravity equation, allowing for the consideration of how economies of scale and differentiated products influence trade flows. His approach incorporated elements of the new trade theory, which emphasizes economies of scale and product differentiation as drivers of trade, particularly among developed nations with similar economic profiles. [18] further extended the theoretical scope of the gravity model by deriving it within the Heckscher-Ohlin (HO) framework, a staple of classical trade theory that attributes trade patterns to countries’ factor endowments. Deardorff’s contribution illustrated that the gravity equation could be consistent with the HO model, particularly under scenarios of frictionless trade or the presence of trade barriers. This integration positioned the gravity model within both the neoclassical and the new trade theory paradigms, enhancing its applicability across diverse theoretical perspectives.

### 5.1. Assumptions of the augmented gravity model

The augmented gravity model of international trade builds on the original Tinbergen [28] specification and the theoretically grounded framework of Anderson and van Wincoop [34] by incorporating additional variables such as common language, colonial links, regional trade agreements, infrastructure, institutional quality, and cultural proximity. Despite these extensions, it retains several core assumptions. First, bilateral trade flows are positively proportional to the economic masses of the trading partners (usually proxied by GDP) and inversely proportional to bilateral trade costs, with geographic distance serving as the primary observable proxy for such costs. Second, economic size is treated as exogenous or weakly endogenous in the short to medium run. Third, trade costs are assumed to be log-linear in observable factors (distance, contiguity, language, RTAs, etc.) plus an error term. Fourth, the model accounts for multilateral resistance terms—either through country fixed effects, remoteness indices, or structural estimation of outward and inward price indices [34]—to avoid omitted-variable bias. Fifth, consumer preferences are homothetic with constant elasticity of substitution (CES) across varieties, and goods are differentiated by origin (Armington assumption). Sixth, markets operate under perfect or monopolistic competition with iceberg trade costs. Finally, the augmenting variables are typically assumed exogenous or appropriately instrumented when endogeneity arises [35–39]. These assumptions preserve theoretical consistency while enhancing empirical applicability

The conceptual framework for this study links the ASEAN–India Free Trade Agreement (AIFTA, implemented in 2010) to bilateral trade flows through a sequence of mechanisms. First, AIFTA reduces tariffs and harmonizes certain trade measures between India and ASEAN countries, lowering bilateral policy barriers. Second, tariff reductions and related facilitation measures lower bilateral trade costs — both direct (tariffs) and indirect (procedural delays, compliance costs) — and also reduce elements of Multilateral Trade Resistance (MTR) faced by exporters/importers. Third, lower trade costs increase the volume of bilateral trade, ceteris paribus. In the gravity model, these mechanisms are operationalized by including tariff measures and an FTA dummy (post-2010) as key explanatory variables, while controlling for standard gravity determinants (GDP of both partners, population, distance) and institutional variables (democracy, globalization). Country and time fixed effects are used to capture unobserved multilateral resistance and global shocks respectively.

Recent studies have further refined the gravity model by employing panel data techniques, which offer several advantages over traditional cross-sectional approaches. Panel data allows for the inclusion of time-series dimensions, enabling researchers to control for country-pair-specific factors that might otherwise bias results. For example, panel data could control for unobserved heterogeneity, capturing effects that vary over time and between trading partners. Additionally, recent developments in the gravity model of trade often incorporate variables such as a common language between trading partners, shared borders, and trade agreements. In this study, we have also included the levels of democracy in India and ASEAN trading partners to account for non-economic factors that influence trade flows. The present paper employed four different models, estimated with and without controls, for exports and imports. Hence, the equation 2 is modified as follows.


$$\begin{array}{cc} {EX}_{it}=\; & \alpha +\beta_{\text{1}}{(GDP}_{it})+{\beta_{\text{2}}(GDP}_{jt})+\beta_{\text{3}}\left({PoP}_{it}\right)+\beta_{\text{4}}\left({PoP}_{jt}\right)+\beta_{\text{5}}\left({TA}_{ijt}\right)+\beta_{\text{6}}\left({Dist}_{ij}\right)\\ & \;\;+\beta_{\text{7}}\left({CC}_{ij}\right)+\beta_{\text{8}}\left({CL}_{ij}\right)+\beta_{\text{9}}\left({CB}_{ij}\right){+\beta }_{\text{1}\text{0}}\left({FTA}_{ij}\right)+\mu_{ijt} \end{array}$$ (3)

$$\begin{array}{cc} {EX}_{it}=\; & \alpha +\beta_{\text{1}}{(GDP}_{it})+{\beta_{\text{2}}(GDP}_{jt})+\beta_{\text{3}}\left({PoP}_{it}\right)+\beta_{\text{4}}\left({PoP}_{jt}\right)+\beta_{\text{5}}\left({TA}_{ijt}\right)+\beta_{\text{6}}\left({Dist}_{ij}\right)\\ & \;\;+\beta_{\text{7}}\left({CC}_{ij}\right)+\beta_{\text{8}}\left({CL}_{ij}\right)+\beta_{\text{9}}\left({CB}_{ij}\right)+\beta_{\text{1}\text{0}}\left({TO}_{ijt}\right)+\beta_{\text{1}\text{1}}\left({LDI}_{it}\right)+\beta_{\text{1}\text{2}}\left({LDI}_{jt}\right)\\ & \;\;+\beta_{\text{1}\text{3}}\left({GI}_{ijt}\right){{+\beta }_{\text{1}\text{4}}\left({FTA}_{ij}\right)+\mu }_{ijt} \end{array}$$ (4)

$$\begin{array}{cc} {IM}_{it}= & \;\alpha +\beta_{\text{1}}{(GDP}_{it})+{\beta_{\text{2}}(GDP}_{jt})+\beta_{\text{3}}\left({PoP}_{it}\right)+\beta_{\text{4}}\left({PoP}_{jt}\right)+\beta_{\text{5}}\left({TA}_{ijt}\right)+\beta_{\text{6}}\left({Dist}_{ij}\right)\\ & \;+\beta_{\text{7}}\left({CC}_{ij}\right)+\beta_{\text{8}}\left({CL}_{ij}\right)+\beta_{\text{9}}\left({CB}_{ij}\right){+\beta }_{\text{1}\text{0}}\left({FTA}_{ij}\right)+\mu_{ijt} \end{array}$$ (5)

$$\begin{array}{cc} {IM}_{it}=\; & \alpha +\beta_{\text{1}}{(GDP}_{it})+{\beta_{\text{2}}(GDP}_{jt})+\beta_{\text{3}}\left({PoP}_{it}\right)+\beta_{\text{4}}\left({PoP}_{jt}\right)+\beta_{\text{5}}\left({TA}_{ijt}\right)+\beta_{\text{6}}\left({Dist}_{ij}\right)\\ & \;\;+\beta_{\text{7}}\left({CC}_{ij}\right)+\beta_{\text{8}}\left({CL}_{ij}\right)+\beta_{\text{9}}\left({CB}_{ij}\right)+\beta_{\text{1}\text{0}}\left({TO}_{ijt}\right)+\beta_{\text{1}\text{1}}\left({LDI}_{it}\right)+\beta_{\text{1}\text{2}}\left({LDI}_{jt}\right)\\ & \;\;+\beta_{\text{1}\text{3}}\left({GI}_{ijt}\right){{+\beta }_{\text{1}\text{4}}\left({FTA}_{ij}\right)+\;\mu }_{ijt} \end{array}$$ (6)

where i = India and j shows the ASEAN partners countries; t donates the years (1990–2022)Equations (1)–(4) represent the augmented gravity models used to estimate India’s bilateral exports (EX) and imports (IM) with ASEAN partner countries. In these models, idenotes India, jrefers to the ASEAN partner country, and tindicates the time period (1990–2022). The dependent variables $EX_{it}$and $IM_{it}$capture India’s export flows to, and import flows from, each ASEAN country in year t.

The explanatory variables include the economic size of the trading partners—represented by their respective GDP levels $\left(GDP_{it},GDP_{jt}\right)$—and demographic size, captured through population $\left(PoP_{it},PoP_{jt}\right)$. Trade policy variables include the applied tariff rate ($TA_{ijt}$), trade openness ($TO_{ijt}$), and the FTA dummy ($FTA_{ij}$), which equals 1 for years after India–ASEAN FTA implementation and 0 otherwise.

Geographical and cultural proximity are measured using the bilateral distance between countries ($Dist_{ij}$), common currency ($CC_{ij}$), common language ($CL_{ij}$), and common border ($CB_{ij}$). Institutional and globalization effects are incorporated through the level of democracy in India and partner countries $\left(LDI_{it},LDI_{jt}\right)$and the degree of globalization ($GI_{ijt}$). The error term $\mu_{ijt}$captures unobserved bilateral factors affecting trade.

Together, these variables allow the model to quantify how economic size, population, trade costs, institutional characteristics, and the India–ASEAN FTA influence bilateral trade flows over time.

The term $\theta_{i}$ represents the country-fixed effect, which accounts for factors such as infrastructure, Multilateral Trade Resistance (MRT), and other unique characteristics specific to each country [40,41]. Similarly, $\gamma_{t}$ denotes the time-fixed effect, capturing variations in trade caused by fluctuations in economic cycles and global trade disruptions, including natural shocks [40–42]. Lastly, $\mu_{ijt}\;$ the idiosyncratic error term, which changes across different countries and over time. Traditional panel data estimations can lead to biased results if cross-sectional dependence, serial correlation, and heteroscedasticity are present [40]. To address these issues, this study uses Feasible Generalized Least Squares (FGLS) as the main estimation technique. FGLS is well-suited for handling panel data with cross-sectional dependence, serial correlation, and heteroscedasticity [43,44]. The FGLS estimation is based on the model specified in Equations (1–4).

## 6. Results and discussions

Before estimating the panel models, three preliminary tests were performed. These include the Wooldridge test for checking autocorrelation [45], the modified Wald test (or Breusch-Pagan test) for detecting heteroscedasticity [46], and Pesaran’s test for cross-sectional dependence [47] . The test results show evidence of both serial autocorrelation and heteroscedasticity under fixed-effect specifications (Table 4). Therefore, in line with methods used in earlier studies [44], the models are estimated using the Feasible Generalized Least Squares (FGLS) approach. The diagnostic tests conducted to validate the model include assessments for multicollinearity, heteroscedasticity, and autocorrelation, indicating no significant statistical issues. The Variance Inflation Factor (VIF) values are all below the threshold of 10, suggesting an absence of multicollinearity. The Breusch-Pagan test yields a p-value above 0.05, indicating homoscedasticity, meaning that the error terms exhibit constant variance. Additionally, the Wooldridge test for autocorrelation shows a p-value exceeding 0.05, confirming that no serial correlation exists within the residuals. Overall, these diagnostic test results suggest that the model is statistically robust and reliable for further interpretation and analysis.

**Table 4:** Results of Diagnostic Tests.

Test	Method	Null hypothesis	Test Statistic	P-value
Heteroskedasticity	Breusch-Pagan test	Residuals are homoscedastic	419.92***	0.000
Cross Sectional Dependency	Pesaran CD test	Residuals are not correlated	2.933***	0.003
Serial Correlation	Breusch-Godfrey Lagrange multiplier test	No serial correlation	4.061*	0.074

Source: Authors’ calculations.

### 6.1. Impact of India- ASEAN trade agreement on exports flows

**Table 5:** Results of Pooled OLS and GLS: Exports.

Variables	Pooled OLS: Exports		GLS method: Exports	
	Without Control	With Control	Without Control	With Control
	Pooled OLS	Pooled OLS	GLS	GLS
Log (GDP_India)	−0.93***	0.32	−0.71***	0.32
	(0.26)	(0.25)	(0.19)	(0.24)
Log (GDP_Partner_ASEAN)	1.17***	1.01***	1.16***	1.01***
	(0.06)	(0.07)	(0.05)	(0.07)
Log (Popoulation_India)	7.93***	2.76**	5.85***	2.76**
	(1.19)	(1.12)	(0.86)	(1.10)
Log (Population_Partner_ASEAN)	0.37***	0.41***	0.33***	0.41***
	(0.07)	(0.06)	(0.05)	(0.06)
Log (Tariff)	−0.01	−0.06*	−0.05*	−0.06*
	(0.04)	(0.03)	(0.03)	(0.03)
Log (Distance_Country to Country)	−2.20***	−0.55**	−2.00***	−0.55**
	(0.25)	(0.26)	(0.17)	(0.26)
Common colony	1.83***	0.74***	1.66***	0.74***
	(0.20)	(0.19)	(0.14)	(0.19)
Common border	−1.42***	0.93***	−1.17***	0.93***
	(0.31)	(0.35)	(0.24)	(0.34)
Common language	0.73***	0.26**	0.61***	0.26**
	(0.13)	(0.12)	(0.09)	(0.12)
Log (Trade Openness)		0.82***		0.82***
		(0.10)		(0.10)
Log (Level of Democracy_India)		1.36***		1.36***
		(0.26)		(0.26)
Log (Level of Democracy_Partner)		−0.18***		−0.18***
		(0.07)		(0.07)
Log (Globalization)		1.06***		1.06***
		(0.35)		(0.34)
FTA Dummy (0 = No FTA, 1 = FTA)		1.08***		1.09***
		(0.13)		(0.06)
Constant	−164.54***	−82.49***	−123.57***	−82.49***
	(23.34)	(21.61)	(16.73)	(21.15)
Time Effect	Yes	Yes	Yes	Yes
Country fixed effects	Yes	Yes	Yes	Yes
Observations	330	330	330	330
R-squared	0.94	0.96		

Standard errors in parentheses, *** p < 0.01, ** p < 0.05, * p < 0.1.

Table 5 presents the results of the Pooled OLS regression, analyzing the impact of various factors on India’s exports to ASEAN countries. The first column shows the results without control variables, while the second column includes control variables.

In the baseline model without control variables, India’s GDP exhibits a negative and statistically significant impact on export levels (coefficient: −0.93), suggesting that as India’s economy grows, its reliance on exports may diminish, potentially due to a stronger domestic market orientation. Conversely, the GDP of ASEAN partner countries exert a positive and highly significant influence on exports (coefficient: 1.17), indicating that economic expansion in partner countries correlates positively with India’s export volume, likely due to increased demand in these markets. India’s population shows a robust positive effect on exports (coefficient: 7.93), suggesting that a larger domestic market is positively associated with export capacity, potentially due to economies of scale or enhanced production capabilities. Similarly, the population of ASEAN partner countries has a positive and significant effect (coefficient: 0.37), highlighting the role of larger foreign markets in sustaining higher export levels. The tariff coefficient is negative but statistically insignificant (−0.01), indicating that tariff variations have not substantially affected export flows in this model. The coefficient for geographical distance between India and its trading partners is negative and significant (−2.20), affirming that greater physical separation tends to inhibit trade, possibly due to increased transportation costs and logistical barriers. Shared colonial history and a common language have significant positive effects, suggesting that historical and linguistic ties facilitate trade. Interestingly, the common border variable is negative, suggesting that geographical proximity without strong institutional trade frameworks may not necessarily enhance exports.

When control variables are introduced, the effects shift in key areas. India’s GDP effect loses significance (coefficient: 0.32), while the GDP of ASEAN countries remain a significant positive determinant (coefficient: 1.01), reinforcing the importance of partner economic size. India’s population and the populations of ASEAN partners maintain positive effects on exports, albeit with slightly reduced magnitudes. Tariffs now exhibit a small but statistically significant negative effect (−0.06), suggesting that tariff reductions marginally enhance trade. The effect of distance decreases notably (−0.55), indicating that some deterrence effects of distance are mitigated when institutional and policy variables are included.

The inclusion of controls shows that shared colonial history, common language, and common border variables all significantly enhance trade. Additionally, trade openness (coefficient: 0.82), India’s level of democracy (coefficient: 1.36), and globalization (coefficient: 1.06) exhibit positive and significant impacts on exports, suggesting that institutional and policy factors play critical roles in facilitating trade flows. Conversely, the democracy level in ASEAN partner countries shows a minor but negative effect (−0.18), which may reflect varying political dynamics across ASEAN economies that influence bilateral trade.

In the GLS model without control variables, India’s GDP exerts a statistically significant negative influence on export levels (coefficient: −0.71), suggesting that domestic economic growth may shift India’s focus towards internal markets, potentially reducing export dependence. Conversely, the GDP of ASEAN partner countries is positively associated with India’s exports (coefficient: 1.16), indicating that economic expansion in partner economies stimulates demand for Indian exports, consistent with established gravity model predictions. India’s population exhibits a substantial positive impact on exports (coefficient: 5.85), underscoring the relationship between a larger domestic market and enhanced export capabilities, possibly due to production scaling or increased market capacity. The population of ASEAN partner countries also positively affects exports (coefficient: 0.33), suggesting that larger ASEAN markets correlate with increased trade volumes. While the coefficient for tariffs is slightly negative (−0.05) and significant at the 10% level, suggesting a modest inhibitory effect on exports, geographical distance between India and ASEAN partners shows a significant negative impact (−2.00), reflecting the expected friction associated with physical separation. Historical and linguistic ties, such as shared colonial history (1.66) and common language (0.61), significantly enhance trade, indicating the importance of familiarity and mutual understanding in trade relations. Notably, the shared border variable exhibits a negative coefficient (−1.17), suggesting that geographical proximity may not directly facilitate exports without strong institutional support.

Upon incorporating control variables in the GLS model, the impact of India’s GDP on exports becomes statistically insignificant (coefficient: 0.32), while ASEAN partners’ GDP maintains its positive and significant effect on exports (coefficient: 1.01). The coefficients for population in both India (2.76) and ASEAN partners (0.41) decrease but remain positive and statistically significant, underscoring the role of demographic size as a persistent determinant of export levels. The negative impact of distance diminishes notably (coefficient: −0.55), indicating that additional variables, such as institutional factors, may partially offset distance-related trade barriers.

Key institutional factors emerge as significant determinants of trade performance. Trade openness (coefficient: 0.82), India’s democracy level (1.36), and globalization (1.06) display positive and statistically significant associations with exports, underscoring the relevance of liberalized trade policies, democratic stability, and integration with global systems in fostering trade. In contrast, the democracy level of partner countries has a slight negative effect on exports (−0.18), which may reflect diverse political-economic contexts within ASEAN that shape bilateral trade interactions differently.

The GLS findings, when interpreted alongside Pooled OLS results, reveal the critical roles of economic scale, demographic factors, and institutional variables in driving India’s exports to ASEAN nations. Both models highlight that factors like trade openness and globalization significantly bolster export volumes, while the complex interplay of economic, geographic, and institutional determinants reveals the nuanced dynamics governing India-ASEAN trade relations. In both econometric models, FTA dummy is showing positive impact on exports means FTA plays an important role in Export between India and ASEAN.

### 6.2. Impact of India- ASEAN free agreement on indian imports

The Pooled Ordinary Least Squares (OLS) regression results, summarized in Table 6, investigate the impact of the India-ASEAN Free Trade Agreement (AIFTA) on India’s import dynamics. In the baseline model without controls, India’s GDP shows a positive and statistically significant relationship with imports (coefficient: 1.07), indicating that as India’s economy expands, its demand for imports increases proportionately. Similarly, the GDP of ASEAN partner countries positively and significantly impact Indian imports (coefficient: 1.27), suggesting that economic growth in ASEAN countries fosters a conducive environment for import demand from India, likely due to increased consumption and production needs.

**Table 6:** Results of Imports OLS Model and GLS Model.

Variables	Pooled OLS: Imports		GLS method: Imports	
	Without Control	With Control	Without Control	With Control
	Pooled OLS	Pooled OLS	GLS	GLS
Log (GDP_India)	1.07**	1.36**	1.07**	0.75***
	(0.50)	(0.55)	(0.49)	(0.28)
Log (GDP_Partner_ASEAN)	1.27***	1.60***	1.27***	0.89***
	(0.12)	(0.16)	(0.12)	(0.14)
Log (Popoulation_India)	−0.72	−0.50	−0.72	1.41
	(2.29)	(2.49)	(2.25)	(1.40)
Log (Population_Partner_ASEAN)	0.66***	0.52***	0.66***	0.68***
	(0.13)	(0.13)	(0.13)	(0.18)
Log (Tariff)	−0.30***	−0.31***	−0.30***	−0.06***
	(0.07)	(0.07)	(0.07)	(0.02)
Log (Distance_Country to Country)	−0.97**	−0.33**	−0.97**	−0.87
	(0.47)	(0.58)	(0.47)	(0.63)
Common colony	3.12***	2.65***	3.12***	2.73***
	(0.38)	(0.43)	(0.38)	(0.46)
common border	−0.53	−0.40	−0.53	−0.34
	(0.60)	(0.77)	(0.59)	(0.89)
Common language	−0.47*	−0.23	−0.47**	−0.13
	(0.24)	(0.27)	(0.24)	(0.33)
Log (Trade Openness)		0.18		0.19
		(0.23)		(0.12)
Log (Level of Democracy_India)		2.16***		0.85**
		(0.58)		(0.43)
Log (Level of Democracy_Partner)		−0.77***		−0.06
		(0.15)		(0.10)
Log (Globalization)		−0.33		0.93
		(0.78)		(0.71)
FTA Dummy (0 = No FTA, 1 = FTA)		2.09**		2.08***
		(0.17)		(0.06)
Constant	−15.42	−32.49	−15.42	−53.50**
	(44.88)	(47.87)	(44.19)	(26.75)
Time Effect	Yes	Yes	Yes	Yes
Country fixed effects	Yes	Yes	Yes	Yes
Observations	330	330	330	330
R-squared	0.85	0.87		

Standard errors in parentheses, *** p < 0.01, ** p < 0.05, * p < 0.1.

Population effects present a nuanced picture: while India’s population coefficient is negative (−0.72) and not statistically significant, ASEAN countries’ population has a positive and significant effect on Indian imports (coefficient: 0.66). This implies that a larger population in ASEAN nations is positively correlated with higher import levels from India, possibly due to expanded market demand within these economies.

The analysis reveals that tariff levels play a crucial restrictive role in trade, with negative and significant coefficients in both models (−0.30 and −0.31), signifying that higher tariffs directly reduce import volumes. The geographical distance between India and ASEAN partners, as expected, exerts a negative influence on imports, with the effect declining in magnitude and significance in the model with controls (coefficients: −0.97 and −0.33), indicating that proximity remains a relevant factor in facilitating trade but can be moderated by other factors when controls are introduced. Historical connections appear to enhance trade ties: a common colonial history is positively associated with imports in both models (coefficients: 3.12 and 2.65), underscoring the role of historical familiarity in sustaining trade relations. The influence of common border and language variables, however, is mixed. The common border variable shows an insignificant impact on imports, while common language surprisingly demonstrates a negative relationship with imports in the uncontrolled model (coefficient: −0.47). This suggests a complex interaction where linguistic commonalities may not uniformly translate into trade facilitation within the AIFTA context, possibly due to regional heterogeneities and divergent economic structures.

Upon incorporating control variables, further institutional factors emerge as significant determinants. India’s democracy level exhibits a robust positive effect on imports (coefficient: 2.16), implying that democratic governance in India supports greater openness to trade. Contrarily, the democracy level in ASEAN countries negatively impacts imports from India (coefficient: −0.77), indicating that lower democratic levels within partner countries may limit bilateral trade engagement. Meanwhile, other controls, including trade openness and globalization, do not display significant effects on imports, suggesting their relatively minor role in shaping import behavior within this specific bilateral trade framework.

3 presents the Generalized Least Squares (GLS) regression results that assess the impact of the India-ASEAN Free Trade Agreement (AIFTA) on India’s import flows. The log of India’s GDP shows a positive and statistically significant effect on imports (coefficient: 1.07) in the model without controls, indicating that increases in India’s economic size are associated with higher import levels. Similarly, the GDP of ASEAN partner countries also has a positive and significant effect (coefficient: 1.27), suggesting that economic expansion within ASEAN countries bolsters imports from India. When controls are added, the coefficients for both India’s GDP and ASEAN partners’ GDP decrease to 0.75 and 0.89, respectively, although the relationship remains significant, highlighting a stable relationship between economic size and import levels. The impact of population dynamics on imports presents a more complex picture. India’s population variable is insignificant in the uncontrolled model (coefficient: −0.72) but becomes positive and significant with controls (coefficient: 1.41), suggesting that other institutional or economic variables may mediate the role of India’s population in influencing import demand. Conversely, the population of ASEAN partner countries consistently shows a significant positive impact on imports in both models (coefficients: 0.66 and 0.68), reflecting that larger ASEAN populations correlate with increased import activity from India.

Tariffs demonstrate a strong negative association with imports in both models, though the effect diminishes slightly when controls are introduced (coefficients: −0.30 and −0.06). This negative relationship underscores the restrictive impact of tariffs on import flows, while the reduction in magnitude with controls suggests that other factors, such as trade liberalization or economic policies, may mitigate the tariff effect to some extent. Distance, as expected, has a negative effect on imports, although its significance decreases in the controlled model, indicating that physical distance remains a trade barrier but may be counterbalanced by other trade-enabling factors. Historical connections are shown to have a robust impact on trade: common colonial ties are positively and significantly associated with imports in both models (coefficients: 3.12 and 2.73), suggesting that colonial legacies foster sustained trade relationships. However, the effects of a common border and language are not significant, implying that while these factors can facilitate trade, they may not be primary drivers within the India-ASEAN trade framework. Institutional factors emerge as influential under the controlled model. India’s level of democracy has a positive and significant impact on imports (coefficient: 0.85), indicating that democratic governance in India is associated with greater openness to trade. Conversely, the democracy level in ASEAN countries does not show a significant impact on imports (coefficient: −0.06), suggesting that the governance structure of ASEAN partners does not strongly influence India’s import levels under AIFTA. In both econometric models, FTA dummy is showing positive impact on imports means FTA plays an important role in Import between India and ASEAN.

## 7. Conclusions and policy implications

This study provides examine the effects of the India-ASEAN Free Trade Agreement (AIFTA) on India’s export and imports by panel data augmented gravity model of trade. The analysis suggests that while India’s GDP is negatively associated with exports, the economic growth of ASEAN countries is positively linked to India’s export. Population metrics further emphasize the significance of demographic scale, demonstrating how the larger populations of both India and ASEAN countries enhance their trade potential by providing a broader consumer base and greater market opportunities. Additionally, our estimated results shows that the significant negative impacts of distance and tariffs on trade flows illustrate the constraining effects of logistical barriers and restrictive trade policies on market access and competitiveness. Regarding imports, findings show a strong positive association with the GDP of both India and its ASEAN partners, affirming the importance of economic size in trade facilitation. The influence of historical connections, particularly through shared colonial history, also emerges as a vital factor, reflecting the longstanding economic ties that foster trade. However, the effects of common borders and language appear mixed, suggesting that while these factors can support trade, they may play a more secondary role. Institutional factors, particularly the positive impact of India’s democratic governance on imports, further indicate that governance structures can shape trade outcomes under AIFTA. Furthermore, the consistently positive and statistically significant coefficient of the FTA dummy across all econometric specifications confirms that the India–ASEAN Free Trade Agreement has had a clear trade-enhancing effect. The agreement has substantially increased both India’s exports to and imports from ASEAN countries, demonstrating that tariff liberalization and deeper regional integration have effectively strengthened bilateral trade ties.

These findings offer several key policy implications. First, improving trade-related infrastructure and streamlining tariff structures can reduce trade barriers and enhance the gains from AIFTA. Developing efficient logistical networks and minimizing regulatory costs will be critical to fostering smoother, more accessible trade channels between India and ASEAN countries. Second, leveraging people-to-people exchanges through educational partnerships, cultural programs, and diplomatic ties could deepen historical connections and promote a more cohesive regional trade environment. Finally, policymakers should recognize the importance of governance and democratic values in trade engagements. Ensuring that trade agreements reflect democratic principles may contribute to more equitable and inclusive trade relationships, promoting long-term stability and mutual benefit across the region.

## Supporting information

S1 Data Data. (ZIP)

S2 Appendix Appendix A. (DOCX)
