# Supplementary material for: The impact of India-ASEAN free trade agreement on trade flows: An application of augmented gravity model
Source: PLoS One. 2026 Jun 3;21(6):e0350036. doi: 10.1371/journal.pone.0350036 (PMC13232826; doi:10.1371/journal.pone.0350036)
Supplement: S1 Data — (ZIP) [file pone.0350036.s001.zip › Data/Tables.docx]

**Table 2.1. India’s Exports to ASEAN Member Countries- 1990-2022** *(values in million US$)*

| **Year** | **Brunei** | **Cambodia** | **Indonesia** | **Lao** | **Malaysia** | **Myanmar** | **Philippines** | **Singapore** | **Thailand** | **Viet Na** | **ASEAN** |
| --- | --- | --- | --- | --- | --- | --- | --- | --- | --- | --- | --- |
| 1990 | 0.669 | 0.136 | 108.132 | 0.030 | 149.212 | 1.851 | 28.028 | 322.273 | 244.250 | 16.957 | 871.538 |
| 1995 | 7.221 | 2.131 | 660.383 | 0.310 | 391.729 | 30.084 | 143.756 | 889.522 | 471.230 | 123.985 | 2720.352 |
| 2000 | 3.057 | 7.886 | 390.371 | 3.506 | 530.951 | 43.185 | 174.192 | 787.048 | 525.237 | 195.393 | 2660.827 |
| 2005 | 4.395 | 21.350 | 1390.066 | 6.540 | 1143.775 | 117.246 | 482.110 | 5427.555 | 1059.267 | 633.465 | 10285.769 |
| 2010 | 21.239 | 61.046 | 4557.083 | 8.180 | 3555.312 | 272.579 | 801.607 | 9066.230 | 2139.581 | 2475.596 | 22958.453 |
| 2015 | 30.392 | 145.346 | 2868.880 | 51.264 | 4892.061 | 859.973 | 1304.346 | 7805.084 | 3113.562 | 5357.213 | 26428.121 |
| 2020 | 60.051 | 144.043 | 4363.742 | 27.874 | 6194.006 | 837.624 | 1416.026 | 8295.020 | 3777.064 | 4500.549 | 29615.999 |
| 2022 | 68.810 | 227.355 | 9866.614 | 16.900 | 7190.535 | 765.164 | 2160.846 | 11830.795 | 6039.395 | 5880.778 | 44047.192 |

Source: WITS.

**Table 2.2. India’s Imports from ASEAN Member Countries- 1990-2022** *(values in million US$)*

| **Year** | **Brunei** | **Cambodia** | **Indonesia** | **Lao** | **Malaysia** | **Myanmar** | **Philippines** | **Singapore** | **Thailand** | **Vietnam** | **ASEAN** |
| --- | --- | --- | --- | --- | --- | --- | --- | --- | --- | --- | --- |
| 1990 | 0.004 | 0.663 | 80.209 | 0.263 | 528.868 | 91.604 | 4.497 | 490.982 | 63.758 | 57.189 | 1318.036 |
| 1995 | 0.036 | 0.341 | 460.089 | NA | 896.819 | 161.989 | 21.412 | 853.340 | 169.299 | 15.456 | 2578.780 |
| 2000 | 0.184 | 1.270 | 985.839 | NA | 1311.030 | 178.499 | 68.256 | 1401.560 | 339.212 | 13.541 | 4299.391 |
| 2005 | 0.832 | 0.425 | 3018.949 | 0.072 | 2435.996 | 489.162 | 203.196 | 3159.416 | 1196.597 | 127.378 | 10632.024 |
| 2010 | 207.130 | 7.638 | 9695.329 | 20.117 | 5995.904 | 1122.147 | 394.450 | 7263.136 | 3940.819 | 993.512 | 29640.184 |
| 2015 | 607.783 | 42.989 | 13902.025 | 142.955 | 9559.921 | 1016.301 | 518.188 | 7395.998 | 5650.145 | 2680.089 | 41516.392 |
| 2020 | 422.894 | 38.186 | 12020.795 | 2.075 | 7378.041 | 575.594 | 505.658 | 12306.747 | 5223.762 | 5564.634 | 44038.387 |
| 2022 | 317.254 | 120.435 | 28665.341 | 57.533 | 13542.358 | 1032.316 | 896.446 | 24418.725 | 11250.487 | 9004.743 | 89305.637 |

Source: WITS. Note: NA= Data is not available.

**Table 4.1. Data Sources and Variables Used in the Estimation**

| **Variable** | **Source** | **Expected Sign** |
| --- | --- | --- |
| Export of India to ASEAN | World Integrated Trade Solutions (WITS) |  |
| Import of India from ASEAN | World Integrated Trade Solutions (WITS) |  |
| Log (GDP_India) | World Bank Development Indicators (WDI) | Positive |
| Log (GDP_Partner_ASEAN) | World Bank Development Indicators (WDI) | Positive |
| Log (Popoulation_India) | World Bank Development Indicators (WDI) | Positive |
| Log (Population_Partner_ASEAN) | World Bank Development Indicators (WDI) | Positive |
| Log (Tariff) | World Bank Development Indicators (WDI) | Negative |
| Log (Distance i and j) | CEPII | Negative |
| Common colony | CEPII | Positive |
| Common border | CEPII | Positive |
| Common language | CEPII | Positive |
| Log (Trade Openness) | World Bank Development Indicators (WDI) | Positive |
| Log (Level of Democracy_India) | Varieties of Democracy (V-Dem) | Positive |
| Log (Level of Democracy_Partner) | Varieties of Democracy (V-Dem) | Positive |
| Log (Globalization) | Varieties of Democracy (V-Dem) | Positive |
| FTA Dummy (0 = No FTA, 1 = FTA) | Authors’ construction based on AIFTA implementation (2010 onward) | Positive |

Source: authors compilation

**Table: 5.1 Results of Diagnostic Tests**

| **Test** | **Method** | **Null hypothesis** | **Test Statistic** | **P-value** |
| --- | --- | --- | --- | --- |
| Heteroskedasticity | Breusch-Pagan test | Residuals are homoscedastic | 419.92*** | 0.000 |
| Cross Sectional Dependency | Pesaran CD test | Residuals are not  correlated | 2.933*** | 0.003 |
| Serial Correlation | Breusch-Godfrey Lagrange multiplier test | No serial correlation | 4.061* | 0.074 |

Source: Authors’ calculations.

**Table 5.2. Results of Pooled OLS and GLS: Exports**

| **Variables** | **Pooled OLS: Exports** | **GLS method: Exports** |
| --- | --- | --- |

|  | Without Control | With Control | Without Control | With Control |
| --- | --- | --- | --- | --- |
|  | Pooled OLS | Pooled OLS | GLS | GLS |
| Log (GDP_India) | -0.93*** | 0.32 | -0.71*** | 0.32 |
|  | (0.26) | (0.25) | (0.19) | (0.24) |
| Log (GDP_Partner_ASEAN) | 1.17*** | 1.01*** | 1.16*** | 1.01*** |
|  | (0.06) | (0.07) | (0.05) | (0.07) |
| Log (Popoulation_India) | 7.93*** | 2.76** | 5.85*** | 2.76** |
|  | (1.19) | (1.12) | (0.86) | (1.10) |
| Log (Population_Partner_ASEAN) | 0.37*** | 0.41*** | 0.33*** | 0.41*** |
|  | (0.07) | (0.06) | (0.05) | (0.06) |
| Log (Tariff) | -0.01 | -0.06* | -0.05* | -0.06* |
|  | (0.04) | (0.03) | (0.03) | (0.03) |
| Log (Distance_Country to Country) | -2.20*** | -0.55** | -2.00*** | -0.55** |
|  | (0.25) | (0.26) | (0.17) | (0.26) |
| Common colony | 1.83*** | 0.74*** | 1.66*** | 0.74*** |
|  | (0.20) | (0.19) | (0.14) | (0.19) |
| Common border | -1.42*** | 0.93*** | -1.17*** | 0.93*** |
|  | (0.31) | (0.35) | (0.24) | (0.34) |
| Common language | 0.73*** | 0.26** | 0.61*** | 0.26** |
|  | (0.13) | (0.12) | (0.09) | (0.12) |
| Log (Trade Openness) |  | 0.82*** |  | 0.82*** |
|  |  | (0.10) |  | (0.10) |
| Log (Level of Democracy_India) |  | 1.36*** |  | 1.36*** |
|  |  | (0.26) |  | (0.26) |
| Log (Level of Democracy_Partner) |  | -0.18*** |  | -0.18*** |
|  |  | (0.07) |  | (0.07) |
| Log (Globalization) |  | 1.06*** |  | 1.06*** |
|  |  | (0.35) |  | (0.34) |

| FTA Dummy (0=No FTA, 1=FTA) |  | 1.08*** |  | 1.09*** |
| --- | --- | --- | --- | --- |
|  |  | (0.13) |  | (0.06) |

| Constant | -164.54*** | -82.49*** | -123.57*** | -82.49*** |
| --- | --- | --- | --- | --- |
|  | (23.34) | (21.61) | (16.73) | (21.15) |

| Time Effect | Yes | Yes | Yes | Yes |
| --- | --- | --- | --- | --- |
| Country fixed effects | Yes | Yes | Yes | Yes |

| Observations | 330 | 330 | 330 | 330 |
| --- | --- | --- | --- | --- |
| R-squared | 0.94 | 0.96 |  |  |

Standard errors in parentheses, *** p<0.01, ** p<0.05, * p<0.1

Table 5.2 presents the results of the Pooled OLS regression, analyzing the impact of various factors on India’s exports to ASEAN countries. The first column shows the results without control variables, while the second column includes control variables.

**Table 5.3. Results of Imports OLS Model and GLS Model**

| **Variables** | **Pooled OLS: Imports** | | **GLS method: Imports** | |
| --- | --- | --- | --- | --- |
|  | Without Control | With Control | Without Control | With Control |
|  | Pooled OLS | Pooled OLS | GLS | GLS |
| Log (GDP_India) | 1.07** | 1.36** | 1.07** | 0.75*** |
|  | (0.50) | (0.55) | (0.49) | (0.28) |
| Log (GDP_Partner_ASEAN) | 1.27*** | 1.60*** | 1.27*** | 0.89*** |
|  | (0.12) | (0.16) | (0.12) | (0.14) |
| Log (Popoulation_India) | -0.72 | -0.50 | -0.72 | 1.41 |
|  | (2.29) | (2.49) | (2.25) | (1.40) |
| Log (Population_Partner_ASEAN) | 0.66*** | 0.52*** | 0.66*** | 0.68*** |
|  | (0.13) | (0.13) | (0.13) | (0.18) |
| Log (Tariff) | -0.30*** | -0.31*** | -0.30*** | -0.06*** |
|  | (0.07) | (0.07) | (0.07) | (0.02) |
| Log (Distance_Country to Country) | -0.97** | -0.33** | -0.97** | -0.87 |
|  | (0.47) | (0.58) | (0.47) | (0.63) |
| Common colony | 3.12*** | 2.65*** | 3.12*** | 2.73*** |
|  | (0.38) | (0.43) | (0.38) | (0.46) |
| common border | -0.53 | -0.40 | -0.53 | -0.34 |
|  | (0.60) | (0.77) | (0.59) | (0.89) |
| Common language | -0.47* | -0.23 | -0.47** | -0.13 |
|  | (0.24) | (0.27) | (0.24) | (0.33) |
| Log (Trade Openness) |  | 0.18 |  | 0.19 |
|  |  | (0.23) |  | (0.12) |
| Log (Level of Democracy_India) |  | 2.16*** |  | 0.85** |
|  |  | (0.58) |  | (0.43) |
| Log (Level of Democracy_Partner) |  | -0.77*** |  | -0.06 |
|  |  | (0.15) |  | (0.10) |
| Log (Globalization) |  | -0.33 |  | 0.93 |
|  |  | (0.78) |  | (0.71) |
| FTA Dummy (0=No FTA, 1=FTA) |  | 2.09** |  | 2.08*** |
|  |  | (0.17) |  | (0.06) |
| Constant | -15.42 | -32.49 | -15.42 | -53.50** |
|  | (44.88) | (47.87) | (44.19) | (26.75) |
| Time Effect | Yes | Yes | Yes | Yes |
| Country fixed effects | Yes | Yes | Yes | Yes |
| Observations | 330 | 330 | 330 | 330 |
| R-squared | 0.85 | 0.87 |  |  |

Standard errors in parentheses, *** p<0.01, ** p<0.05, * p<0.1

**Appendix A**

**List of countries included in the estimation of models**

| **Sl. No** | **Country** | **Level of Development** |
| --- | --- | --- |
| 1 | Brunei Darussalam | High income country |
| 2 | Cambodia | Low and middle-income Country |
| 3 | Indonesia | Low and middle-income Country |
| 4 | Lao PDR | Low and middle-income Country |
| 5 | Malaysia | Low and middle-income Country |
| 6 | Myanmar | Low and middle-income Country |
| 7 | Philippines | Low and middle-income Country |
| 8 | Singapore | High income country |
| 9 | Thailand | Low and middle-income Country |
| 10 | Viet Nam | Low and middle-income Country |

Source: Authors creation based on World Bank (WB) income classification.
