# Supplementary material for: The impact of India-ASEAN free trade agreement on trade flows: An application of augmented gravity model
Source: PLoS One. 2026 Jun 3;21(6):e0350036. doi: 10.1371/journal.pone.0350036 (PMC13232826; doi:10.1371/journal.pone.0350036)
Supplement: S2 Appendix — (DOCX) [file pone.0350036.s002.docx]

**Appendix A**

**List of countries included in the estimation of models**

| **Sl. No** | **Country** | **Level of Development** |
| --- | --- | --- |
| 1 | Brunei Darussalam | High income country |
| 2 | Cambodia | Low and middle-income Country |
| 3 | Indonesia | Low and middle-income Country |
| 4 | Lao PDR | Low and middle-income Country |
| 5 | Malaysia | Low and middle-income Country |
| 6 | Myanmar | Low and middle-income Country |
| 7 | Philippines | Low and middle-income Country |
| 8 | Singapore | High income country |
| 9 | Thailand | Low and middle-income Country |
| 10 | Viet Nam | Low and middle-income Country |

Source: Authors creation based on World Bank (WB) income classification.
